# A multipurpose instrument for time-resolved ultra-small-angle and coherent X-ray scattering

**DOI:** 10.1107/S1600576718012748

**Published:** 2018-10-11

**Authors:** Theyencheri Narayanan, Michael Sztucki, Pierre Van Vaerenbergh, Joachim Léonardon, Jacques Gorini, Laurent Claustre, Franc Sever, John Morse, Peter Boesecke

**Affiliations:** a ESRF – The European Synchrotron, 38043 Grenoble, France

**Keywords:** small-angle X-ray scattering, SAXS, ultra-small-angle X-ray scattering, USAXS, X-ray photon correlation spectroscopy, XPCS, time-resolved X-ray scattering

## Abstract

This article presents the main technical features and performance of the upgraded beamline ID02 at the ESRF. The beamline combines different small-angle X-ray scattering techniques in one unique instrument, enabling static and kinetic investigations from ångström to micrometre size scales and time resolution down to the sub-millisecond range. The main component of the instrument is an evacuated detector tube of length 34 m and diameter 2 m. Several different detectors are housed inside a motorized wagon that travels along a rail system, allowing an automated change of the sample–detector distance from about 1 to 31 m as well as selection of the desired detector. For optional combined wide-angle scattering measurements, a wide-angle detector is installed at the entrance cone of the tube. A scattering vector (of magnitude *q*) range of 0.002 ≤ *q* ≤ 50 nm^−1^ is covered with two sample–detector distances and a single-beam setting for an X-ray wavelength of 1 Å. In the high-resolution mode, two-dimensional ultra-small-angle X-ray scattering patterns down to *q* < 0.001 nm^−1^ can be recorded, and the resulting one-dimensional profiles have superior quality as compared to those measured with an optimized Bonse–Hart instrument. In the highest-resolution mode, the beam is nearly coherent, thereby permitting multispeckle ultra-small-angle X-ray photon correlation spectroscopy measurements. The main applications of the instrument include the elucidation of static and transient hierarchical structures, and nonequilibrium dynamics in soft matter and biophysical systems.

## Abstract 2

The technical features and performance of a new instrument for time-resolved ultra-small-angle and coherent X-ray scattering are presented. The instrument enables static and kinetic investigations from ångström to micrometre size scales and time resolution down to the sub-millisecond range. Applications include elucidation of static and transient hierarchical structures in soft matter and biophysical systems.

## 1. Introduction   

Over the past several decades, high-brilliance small-angle X-ray scattering (SAXS) and related techniques have been widely used in the investigation of soft matter and noncrystalline biological systems (Narayanan *et al.*, 2017 ▸; Tuukkanen *et al.*, 2017 ▸). A unique feature of these scattering methods is that they offer simultaneously high spatial and temporal resolution, albeit in reciprocal space. Moreover, scattering experiments can be combined with a variety of thermophysical, rheological and biophysical techniques, thereby further enhancing the structural and dynamical information (Narayanan *et al.*, 2017 ▸; Sakurai, 2017 ▸). Traditionally, SAXS and wide-angle X-ray scattering (WAXS) experiments and ultra-small-angle X-ray scattering (USAXS) experiments have been performed using two separate instruments in tandem (Narayanan *et al.*, 2001 ▸; Ilavsky *et al.*, 2018 ▸). In this case, the USAXS setup involves a Bonse–Hart multi-crystal instrument (Sztucki & Narayanan, 2007 ▸; Ilavsky *et al.*, 2009 ▸). However, pinhole two-dimensional USAXS has some advantages, especially for investigating highly oriented specimens and in time-resolved studies (Yagi & Inoue, 2003 ▸; Shinohara *et al.*, 2007 ▸; Kishimoto *et al.*, 2014 ▸). In addition, high angular resolution and two-dimensional diffraction patterns are essential for performing crystallography at the colloidal scale (Petukhov *et al.*, 2015 ▸).

This article presents the main technical features and performance of the upgraded beamline ID02 at the ESRF. The primary motivation for this beamline project, named time-resolved ultra-small-angle X-ray scattering (TRUSAXS), was to enlarge the applicability of SAXS to relatively unexplored nonequilibrium processes of fundamental and practical interest. Examples range from complexities of hierarchically self-assembled biomimetic systems (Valéry *et al.*, 2011 ▸) to pathways of cellular regulation (Piazzesi *et al.*, 2018 ▸). To make SAXS techniques useful in the investigation of such complex systems, significant enhancements of the range of accessible scattering vector, angular resolution, scattering detectivity and time resolution are required. While improving the performance of high-brilliance SAXS and WAXS techniques, the TRUSAXS instrument offers a unique feature of two-dimensional USAXS and ultra-small-angle X-ray photon correlation spectroscopy (USA-XPCS), allowing one to probe microstructure and dynamics in the same experiment (Möller, Chushkin *et al.*, 2016 ▸). Although USA-XPCS can be performed with a Bonse–Hart instrument (Sztucki & Narayanan, 2007 ▸; Zhang *et al.*, 2011 ▸), multispeckle analysis (Möller, Chushkin *et al.*, 2016 ▸) and time-resolved measurements are required for the investigation of nonequilibrium systems (Dattani *et al.*, 2017 ▸; Möller & Narayanan, 2017 ▸). Having SAXS, WAXS and USAXS in the same setup greatly enhances the exploitation of USAXS in soft-matter and biophysical studies.

The main technical feature of the TRUSAXS instrument is an evacuated detector flight tube (Van Vaerenbergh *et al.*, 2016 ▸) that permits a continuous variation of the sample–detector distance from 0.8 to 30.8 m. Using several sample–detector distances and an X-ray wavelength (λ) of 1 Å, measurements over a *q* range of 10^−3^ ≤ *q* ≤ 8 nm^−1^ can be performed. Here, *q* is the magnitude of the scattering vector given by *q* = (4π/λ)sin(θ/2), with θ the scattering angle. When SAXS and USAXS are combined with WAXS, the setup covers a *q* range of 10^−3^ ≤ *q* ≤ 50 nm^−1^, corresponding to more than four orders of magnitude in reciprocal space dimensions. This broad *q* range together with sub-millisecond time resolution enables a wide range of dynamical studies from the molecular level to the upper limit of the colloidal scale. The use of long collimation distances diminishes the background of the instrument over the conventional SAXS range, thereby allowing studies of extremely dilute systems. To cover a high dynamic range in *q* and scattered intensity, *I*(*q*), several configurations are implemented. In the high-brilliance mode, the flux (in photons s^−1^) is maximized for the highest time resolution and detection capability. In the high-resolution mode, the high angular resolution is obtained with moderate flux. In the coherence mode, the beam is tightly collimated at the expense of flux.

## 2. Technical features   

**Figure 1:**
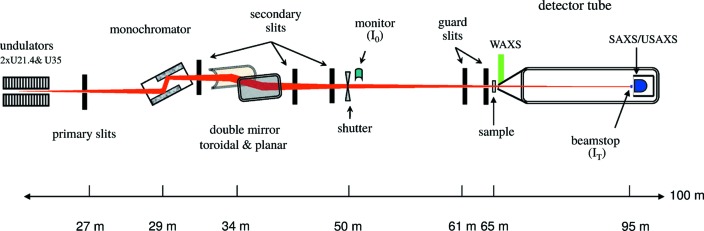
Schematic layout (side view) of the ID02 TRUSAXS beamline depicting the principal components.

The schematic layout of the beamline is depicted in Fig. 1 ▸. The beamline consists of five sections: the source, principal optics, collimation slits, experimental station and detector tube. The relatively long separation between the principal optical components and the sample permits better collimation of the beam, thereby significantly curtailing the background, which is essential for performing USAXS with a two-dimensional detector. The SAXS detectors are enclosed in the wagon within the detector tube and the WAXS detector is mounted outside in air above the entrance cone of the tube.

### 2.1. Undulator source   

The source is located at a high β section of the present storage ring with a full width at half-maximum (FWHM) size and divergence of, respectively, 9 µm and 14 µrad in the vertical and 900 µm and 28 µrad in the horizontal. With the Extremely Brilliant Source (EBS) upgrade, the horizontal beam size and divergence will be reduced to about 56 µm and 17 µrad, respectively, while the vertical parameters remain similar. The X-ray beam is delivered by two phased U21.4 or a single U35 undulator of length 1.6 m each and minimum gap of 11 mm. While U21.4 undulators are primarily used for fixed-energy (*E*) operation (at 12.4 keV and maximum up to 15 keV), the U35 device covers the full energy range of the beamline, 8−20 keV. The front end has a polished diamond window (thickness ∼300 µm) that separates the vacuum of the storage ring from the beamline. The phasing of the two U21.4 undulators increases the flux as compared to two separate devices. The exact gain in flux depends on the opening of the primary slits and it increases with decreasing slit gap. In the standard operation, the primary slits are closed to 0.8 × 0.8 mm and the flux is about 2.3 times larger when two U21.4s are used in tandem as compared to a single U21.4, while this factor increases to 3 for a primary slit opening of 0.3 × 0.3 mm. The combination of two U21.4 undulators and a primary slit aperture of 0.8 × 0.8 mm provides a monochromatic (Δ*E*/*E* ≃ 2 × 10^−4^) flux of about 6 × 10^13^ photons s^−1^ at the sample position. Between 12.4 and 15.2 keV, the U21.4 device delivers more flux than the U35 undulator.

### 2.2. Primary optics   

The main optical elements consist of the primary and secondary slits, the cryogenically cooled monochromator, and the focusing optics. The liquid-nitrogen-cooled monochromator is a generic design using a channel-cut Si 111 crystal (Crystal Scientific Ltd) with a channel width of 5 mm. The channel-cut is similar to a *Z*-cut, which allowed fine polishing and etching of the diffracting surfaces. The channel-cut configuration is not a fixed exit monochromator, and the 5 mm gap results in an increase of beam height by 9.87 mm at 12.4 keV and a height variation of about 50 µm when the energy is increased from 12.4 to 16 keV. The monochromator can withstand a heat load up to 200 W and a power density of the order of 100 W mm^−2^ without significant loss in intensity and broadening of the rocking curve (Zhang *et al.*, 2013 ▸). In addition, an independent tuning of the second diffracting surface is facilitated by a motorized pusher arrangement. The reproducibility of the energy scan is about 0.3 eV.

The choice of focusing optics was guided by several factors. First of all, they should allow observation of the smallest possible scattering angle for a given sample–detector distance and maintain very high temporal stability. Secondly, to preserve the high brilliance the optics should be able to accept the full beam after the monochromator. Thirdly, the change of the sample–detector distance should be relatively easy without needing to refocus the beam. Additionally, the constraints imposed by the building require that the reflected beam is maintained parallel to the incident beam all along the beamline. Finally, the focusing optics should also reject the harmonics in the undulator spectrum which, in order to fulfill the radiation protection regulations, must be below 0.01%. All these conditions are met with a double-mirror scheme consisting of a toroidal mirror and a planar mirror in tandem, which reflects the beam in the horizontal direction. In this scheme, the vertical focusing (sagittal) is less sensitive to the slope error of the mirror (Δβ, the sagittal slope error) since the deviation from ideal focusing (δ*v*) is given by δ*v* = 2sin(α_i_)Δβ, with α_i_ the incident grazing angle. In other words, the sin(α_i_) multiplicative factor diminishes the effect of slope error and enables an ideal source size to be obtained in the vertical direction. However, the horizontal beam size becomes more sensitive to the meridional slope error (Δα) as the deviation (δ*h*) is given by δ*h* = 2Δα. With the present horizontal source parameters, no difference can be observed for a slope error Δα ≃ 0.6 µrad. As a result the double-mirror configuration with the best possible toroidal mirror followed by a fine planar mirror preserves the source properties and maintains high temporal stability. The main optical parameters of the mirrors (Thales SESO SAS) are provided by Van Vaerenbergh *et al.* (2016 ▸).

**Figure 2:**
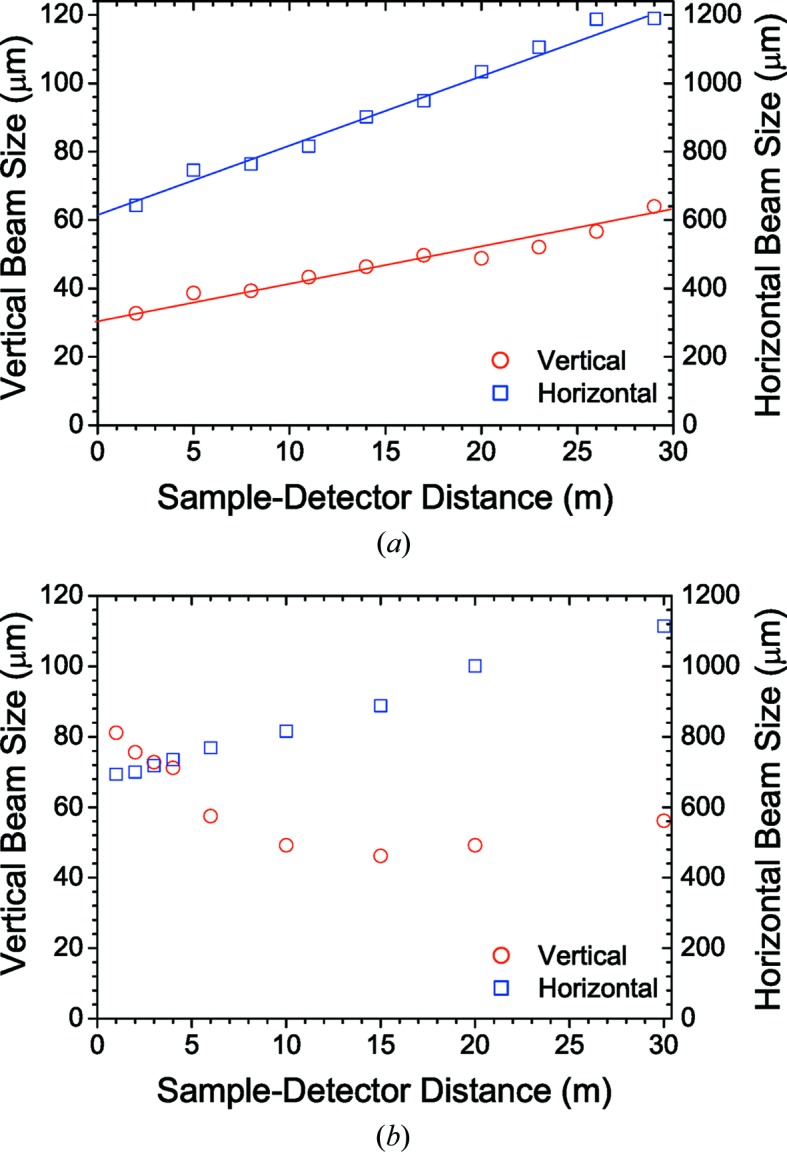
Measured FWHM beam sizes along the detector tube. (*a*) Beam sizes when the mirror inclination is optimized for vertical focus at each distance. (*b*) Variation of beam sizes when the vertical size is optimized for the farthest distance. Note the different scales for horizontal and vertical sizes.

The focusing distance is varied from the sample position to the farthest detector position by changing the inclination of the double-mirror assembly from about 2.6 to 3.5 mrad while maintaining the reflected beam along the same path. When changing the inclination of a toroidal mirror, the horizontal and vertical focal lengths change in opposite directions and consequently the horizontal and vertical beam sizes cannot be simultaneously optimized at a given position. This is not a severe constraint since the beam at the mirror is only 1.5 times larger than at the source horizontally, while it is 50 times larger vertically. Therefore, changing the focal length by inclining the mirror has much less influence horizontally than vertically. The minimum FWHM vertical beam size varies from approximately 30 to 60 µm from the sample to the farthest detector position and the corresponding horizontal beam size changes from 600 to 1200 µm (for a primary slit size of 0.8 × 0.8 mm). Fig. 2 ▸(*a*) shows the beam size measured along the detector tube using a high-resolution camera (beam viewer). The variation of beam size along the detector tube for a fixed focusing is depicted in Fig. 2 ▸(*b*). The measured values are consistent with the expected sizes from the geometrical optics and deviations can be described by ray-tracing calculation in *XOP* (Sánchez del Río & Dejus, 2011 ▸). The enlarge­ment of the vertical beam size from the ideal source size is attributed to a cumulative effect of residual optical aberrations and mechanical vibrations. Further reduction of the beam size is achieved by slit collimation as shown in §3.3.

### 2.3. Secondary optics   

Apart from the main optical elements described above, high-power primary slits (Marion & Zhang, 2004 ▸) are used to reduce the heat load on the monochromator. In addition, these slits are used together with the secondary slits for beam collimation. In the standard configuration, the last two pairs of slits serve as guard slits to curtail the parasitic background from the upstream optics and slits. The last guard slits are scatterless slits (Li *et al.*, 2008 ▸) with hybrid metal/silicon crystal blades (Xenocs SA). When a smaller size than the focused beam is required, the first guard slits are also employed for collimation and only the last slits serve as guard slits. However, the slit collimation is effective only up to a certain aperture size (*ca* 50 and 20 µm along vertical and horizontal directions, respectively), below which the diffraction effect broadens the beam size. The differences are due to different vertical and horizontal transverse coherence lengths. By optimizing primary, secondary and guard slits, a beam cross section of 20 × 20 µm and divergence of ∼1 µrad can be obtained near the sample position, but at the expense of flux (∼10^10^ photons s^−1^). For these small sizes, the beam is highly coherent, producing speckles in the scattered field (Sutton, 2008 ▸).

Alternatively, the horizontal beam divergence could be reduced by using multiple-bounce pseudo-channel-cut crystals (Sztucki *et al.*, 2008 ▸), but again at the expense of flux. As compared to extreme slit collimation, this scheme provides a lower background when relatively weak scattering needs to be measured. For variable beam sizes and coherence applications, the slit collimation is preferred.

### 2.4. Detector tube   

**Figure 3:**
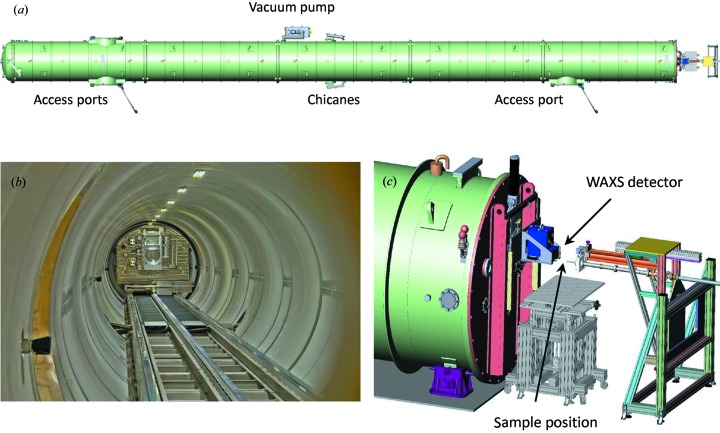
(*a*) Top view of the detector tube and the experiment station. (*b*) Inside view of the detector tube, displaying the wagon, beamstops and rail section. (*c*) Sketch of the experiment station, showing the WAXS detector mounted outside the detector tube near the sample.

The detector flight tube, which has a length of 34 m and a diameter of 2 m, is made of 8 mm thick stainless steel (Added Value Solutions, AVS). Fig. 3 ▸(*a*) shows the top view of the detector tube and the experiment station. Three different SAXS detectors are housed inside a sealed motorized wagon that travels along a rail system within the vacuum tube as depicted in Fig. 3 ▸(*b*). For SAXS/USAXS experiments, this arrangement enables automated change of the sample–detector distance between 0.8 and 30.8 m in a precise and reproducible way (Van Vaerenbergh *et al.*, 2016 ▸). The parasitic lateral movement of the wagon is within ±300 µm over the full stroke of 30 m. The wagon assembly comprises a cabin, a carriage and a translation table which permits further repositioning of the cabin within 100 µm over the 30 m stroke. For normal operation, the interior of the cabin is at atmospheric pressure with vacuum outside. The 100 m^3^ tube volume is evacuated to 10^−2^ mbar (1 Pa) in less than 2 h by an industrial dry screw pump (Edwards GX450). An X-ray-transparent window made of fibrous carbon sheet (thickness ∼400 µm and diameter 240 mm) is glued and bolted onto the front flange of the cabin, and it separates the vacuum in the tube and the atmosphere inside the wagon. The temperature inside the cabin is regulated to 298 K by a heat exchanger system.

Inside the cabin, three detectors, workhorse SAXS/USAXS (Rayonix MX 170 HS), high-resolution USAXS (FReLoN 4M) and time-resolved SAXS (Pilatus 300K, Dectris) detectors, and a high-resolution camera (beam viewer) are installed on a translation table. This arrangement allows positioning of the desired detector behind the X-ray window while shielding the unused detectors. The WAXS detector (Rayonix LX 170 HS) is mounted outside the tube around the entrance cone as depicted in Fig. 3 ▸(*c*), and it can be lifted up by 0.5 m when not in use. A flexible beamstop system is installed in front of the X-ray window in vacuum, which blocks the direct beam (Van Vaerenbergh *et al.*, 2016 ▸). The primary beamstop is made of a lead block (height 2 mm, width 5 mm and thickness 2 mm) with an embedded silicon p–i–n photodiode. The signal from the photodiode is fed to a variable-gain low-noise current amplifier (DLPCA-200, FEMTO) that enables the measurement of transmitted beam intensity (*I*_T_) with high precision. In addition, the beamstop system is equipped with six independent frames with auxiliary beamstops (Van Vaerenbergh *et al.*, 2016 ▸) of circular or rectangular shape with sizes ranging from 1 to 12 mm. They provide a high degree of flexibility in different applications, including fiber diffraction, XPCS and grazing-incidence SAXS. The detector wagon has an absolute position encoder which enables the measurement of translation along the rail with a precision better than 0.1 mm. In addition, the distances from a reference flange in front of the detector tube to the different detectors are precisely calibrated using silver behenate at multiple detector positions. Therefore, the SAXS/USAXS detector distance calibration involves only an accurate measurement of the separation between the sample and the reference flange outside the tube. The WAXS detector distance is usually calibrated with a reference sample, *e.g. para*-bromobenzoic acid (PBBA).

### 2.5. Experiment station   

The experiment station is equipped with essential infrastructure (power outlets, Ethernet sockets, compressed air or nitrogen, chilled water *etc.*) for multipurpose *in situ* experiments. Fig. 3 ▸(*c*) is a sketch of the sample area inside the lead hutch. The main sample table has motorized movements of 600 mm along the horizontal and 350 mm along the vertical with 1 µm precision. This table provides a very stable platform, and relatively large sample environments (maximum 300 kg) can be placed on it. When required, additional fast translation and precision rotation stages are installed on the main sample table. These auxiliary stages facilitate fast vertical movement, rotation along the vertical and tilt along two horizontal axes. The sample environment is placed in air. The vacuum upstream and in the detector tube is isolated by thin high-quality mica windows of thickness ∼15–25 µm, and diameter 10 and 12–16 mm, respectively. The last guard slits are mounted on a sturdy retractable telescopic tube arrangement which enables minimization of the air gap without breaking the vacuum. The experiment control cabin is located just outside the radiation-protected lead hutch. Adequate coaxial cables and serial lines are provided to record signals from sample environments and interface user-supplied instruments with the data acquisition.

### 2.6. Detectors   

In general, SAXS, WAXS and USAXS detectors need to have low intrinsic noise and sufficient sensitivity to provide single-photon detectivity together with high dynamic range and spatial resolution. A high frame rate is essential for time-resolved experiments. In addition, the SAXS detector should have a relatively large active area, preferably without gaps. It is not always possible to meet these specifications with one detector. Therefore, several detectors are utilized to obtain the required characteristics in combination. To meet the high-count-rate, spatial resolution and radiation hardness requirements, fiber-optically coupled CCD-based detectors are employed. For SAXS, a Rayonix detector using split frame transfer CCDs (*e.g.* MX170 HS) meets some of these specifications in terms of overall size (170 × 170 mm), spatial resolution (∼85 µm), sensitivity (the average single-photon level is above the readout noise at 193 K for typical integration times) and frame rate (100 frames per second in 10 × 10 binning with significantly reduced spatial resolution).

For USAXS, a high spatial resolution and dynamic range are critical. Therefore, a specific development was undertaken within the ESRF detector group to assemble a fast readout low-noise (FReLoN) detector (Labiche *et al.*, 2007 ▸) based on a large-area Kodak CCD sensor (KAF-4320) bonded to a thick fiber-optic faceplate (50 × 50 mm) with 1:1 coupling (FReLoN 4M). This detector provides a higher spatial resolution (∼44 µm), high sensitivity (single-photon signal about 5 times above the noise level) and reasonable dynamic range (10^7^ photons mm^−2^). For compatibility and software integration reasons, a Rayonix LX170 HS (85 × 170 mm) was chosen as the WAXS detector. Moreover, this detector has a groove cut at the middle of the long edge that permits the SAXS intensity to pass unobstructed. To complement the CCD detectors, a photon-counting PILATUS 300K detector is installed for highly time-resolved SAXS and XPCS experiments. This detector delivers a maximum frame rate of 400 Hz with a minimum 0.2 ms exposure. Table 1 ▸ provides a summary of the observed technical specifications of the detectors. The Rayonix detectors are operated in the low-noise mode with slightly reduced frame rate compared with the standard mode. The spatial resolution refers to the FWHM point spread function (PSF), which is measured by means of an attenuated beam of 20 µm cross section centered on the pixel and by applying the corresponding Gaussian deconvolution. The pixel detector has a sharp PSF, nearly a box profile with one pixel width, which when approximated by a Gaussian function has an FWHM roughly half the pixel size, as listed in Table 1 ▸. Furthermore, an Eiger 500k detector module (Dinapoli *et al.*, 2011 ▸), which has a pixel size of 75 µm, active area 80 × 40 mm and frame rate up to 22 kHz can be used for XPCS measurements. In the future, it is foreseen that the Rayonix MX170 HS and Pilatus 300K detectors will be replaced by a larger Eiger 4M (Dectris) pixel detector.

Besides the three SAXS detectors, the cabin also houses a high-resolution camera primarily for recording the direct beam profile. This beam viewer is based on a high-resolution scintillator lens coupled to a Basler Gigabit Ethernet CCD camera with a frame rate of about 30 s^−1^. The pixel size is 4.8 µm, corresponding to a Nyquist resolution of 9.6 µm (Martin & Koch, 2006 ▸). In addition to the beamstop photodiode, several beam monitors based on Kapton foil (25 µm) scatterers and p–i–n photodiodes fed to FEMTO current amplifiers (AMP) are installed along the beamline for monitoring the incident beam intensity (*I*_0_) and alignment purposes. These intensity monitors are calibrated using a reference photodiode where the absolute photocurrent and absorption are directly measured.

### 2.7. Fast beam shutter   

The X-ray exposure of the sample is controlled by a fast beam shutter (FBS) that opens only for the duration of the specified acquisition time, and prevents the sample and detectors from continuous exposure during the idle time. The FBS is constructed by a tandem arrangement of two laser shutters (Uniblitz LS2, Vincent Associates) with an aperture of 2 mm. This shutter control has an electronic delay of 0.7 ms after receiving the gate signal and a total shutter transit time of 0.3 ms, corresponding to a combined delay of 1 ms. The exposure time is precisely adjusted by the delay between the gate signals (TTL) to the two shutter controllers. The shutter has a maximum operating frequency of 400 Hz, and sub-millisecond exposure time is obtained by tuning the delay between the two gate signals. This scheme provides a nearly rectangular intensity profile down to 300 µs and even shorter exposure times (triangular) in stroboscopic time-resolved experiments. However, the laser shutter (LS2) does not completely attenuate the X-ray beam at 12.4 keV. Therefore, a lead spot of size ∼2 mm and thickness 0.2 mm is affixed at the position of the X-ray window on the shutter blade without introducing significant additional mechanical inertia.

### 2.8. Data acquisition scheme   

All detectors are integrated into the beamline control software *SPEC* (Certified Scientific Software), *via* LIMA (Library for Image Acquisition) device servers. The LIMA is an ESRF development for the unified control of two-dimensional detectors with the same acquisition, file saving and image processing features (Petitdemange *et al.*, 2018 ▸). Acquired data are directly saved by the LIMA running on the detector control Linux workstations to the central data storage. At any given time, a desired combination of the configured detectors can be selected for image acquisition. The synchronization of the detectors and the fast beam shutter and other accessories is achieved by a time-frame-generator (TFG) unit, which is embedded in a compact PCI module, C216, developed by the Digital Electronics laboratory at the ESRF. The C216 offers a high level of flexibility by combining a TFG, multi-channel scaler (MCS) and voltage-to-frequency converter (VFC) in a single module. The MCS allows simultaneous registering of essential parameters such as incident and transmitted flux, sample temperature, pressure, *etc*. with each two-dimensional image. The TFG can program 4096 frames or 2048 frame pairs with eight independent outputs (TTL). The minimum length of a frame is 100 µs and the maximum is 2^14^ s. The acquisition can be triggered either internally or externally by a TTL signal from the sample environment or user-supplied electronics. The MCS features 16 channels with 32 bit counter depth and the VFC has eight channels. The C216 is used not only for timing and synchronization of detectors and sample environments but also as a counter for normal scans during beamline alignment and sample positioning.

**Figure 4:**
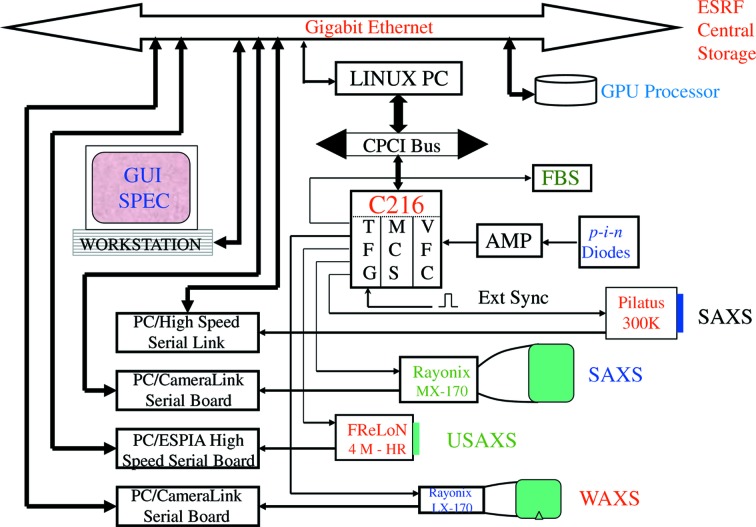
Schematic diagram of the data acquisition using C216 master control. The start of an acquisition sequence can be synchronized with an external event by the external start input and paused by an inhibit signal.

Fig. 4 ▸ schematically depicts the data acquisition scheme based on the C216. In an acquisition sequence, the frame pairs can be equally spaced or varied in a geometric progression or divided into subgroups with different exposure times and waiting times. By varying the length of frames in a geometric progression both the exposure time and the dead time between exposures can be adjusted to match the evolution of scattered intensity and the kinetics under investigation. However, the minimum dead time is determined by the readout rate of a given detector. For CCD detectors, multiple dark images of the same exposure time are taken prior to each acquisition sequence. The delay between the two fast beam shutter controllers is adjusted using a delay generator based on a NIM module (OPIOM). The voltage outputs from the FEMTO current amplifiers of different photodiodes are converted to frequency and counted by the MCS. The offset due to dark current is subtracted from the measured counts and then converted to absolute number of photons.

### 2.9. Data reduction   

Two pipelines are available for online data reduction, which can be configured by the same graphical user interface that is accessible from *SPEC*. Different levels of corrections can be chosen depending on the type of detector (dark image subtraction, flat field division, spatial distortion correction *etc*.). For better performance, detector data and metadata are independently saved to the central data storage by the LIMA servers and *SPEC*, respectively. Subsequently, two-dimensional images are normalized to an absolute intensity scale, azimuthally regrouped and averaged to obtain one-dimensional scattering profiles, while the data acquisition can be continued in parallel. The first data reduction pipeline is based on the online SAXS/WAXS data reduction package (*SPD*) that was developed for the former beamline (Boesecke, 2007 ▸). It offers a high level of flexibility and stores two-dimensional data in the ESRF data format (EDF, with binary data and a comprehensive ASCII header) and one-dimensional data directly in ASCII format. The one-dimensional data can be processed easily by users and read by many data analysis packages. The second pipeline is based on Python fast azimuthal integration (*pyFAI*; Kieffer & Karkoulis, 2013 ▸). This approach makes use of parallel GPU computation, which is a significant improvement for the data reduction in measurements involving high time resolution and throughput. Both two-dimensional and one-dimensional data files are saved in HDF5 containers following the NeXus (Könnecke *et al.*, 2015 ▸) standard.

The HDF5 data as well as EDF image data and ASCII one-dimensional data can be visualized using the advanced version of the MATLAB-based *SAXSutilities* package (http://www.saxsutilities.eu). In addition, conversion routines from HDF5 to EDF (for two-dimensional) and ASCII (for one-dimensional data) are included. *SAXSutilities* enables the display of multiple data sets after background subtraction in different plots used in small-angle scattering. Other features include averaging, rebinning, merging and background subtraction of selected data sets and offline data reduction including error estimation. In addition, routines for masking unusable regions of scattering patterns, determination of the beam center, calibration of the WAXS detector position using PBBA *etc*. are included in this software.

## 3. Technical performance   

**Figure 5:**
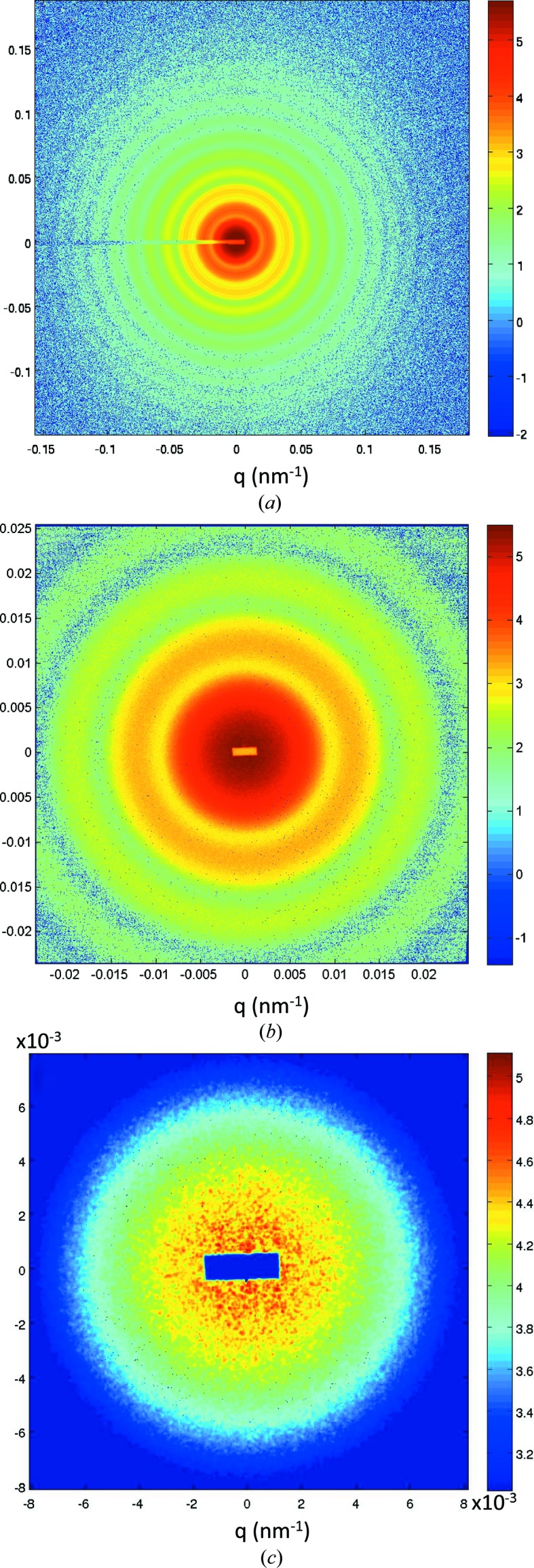
SAXS and USAXS patterns from a dilute colloidal suspension of silica particles (size ∼450 nm) in water measured at a sample–detector distance of 31 m. (*a*) Medium resolution covering a wide *q* range acquired with the Rayonix detector, (*b*) high-resolution pattern recorded by the FReLoN detector and (*c*) speckle pattern measured with the FReLoN detector.

The instrument can be operated in different resolution modes. Fig. 5 ▸ illustrates the different features observed depending on the collimation for the same sample in the USAXS range. The sample consisted of a dilute suspension of silica particles (size ∼450 nm and polydispersity ∼0.05) in water. The intensity modulations arise from the form factor of spherical particles. At medium resolution (Fig. 5 ▸ *a*), one can observe the oscillations in the form factor over a very large *q* range, which is important for the analysis of the morphology of the particles (Narayanan, 2014 ▸). Alternatively, the low-*q* region can be measured with high resolution (Fig. 5 ▸ *b*), which is important to avoid instrumental smearing effects and to accurately determine the structure factor of interactions. Finally, in the coherence mode, one can observe speckles in the scattering pattern (Fig. 5 ▸ *c*) and the analysis of the temporal fluctuations of these intensities yields the equilibrium dynamics of the suspension (Sutton, 2008 ▸).

Radiation damage is a limiting factor in many soft-matter and biophysical investigations. Since samples have to be studied under stringent thermodynamic or physiological conditions, specific solutions are adapted for each system. To avoid damage, the beam size on the sample, the exposure time and the delay between successive exposures are assessed in each case. The focusing and collimation slits are adjusted such that the beam size is maximized on the sample and minimized on the detector. When a small beam spot is required on the sample, the flux, exposure time and delay between exposures are optimized to remain below the damage threshold. The specimen is usually displaced between successive exposures, which in the case of fluid samples is effected by pushing the column using a flow-through capillary cell.

The performance of the detectors depends on how well they are calibrated. The pixel size and spatial distortion of each detector are calibrated using a copper grid with a regular array of holes precisely separated by 5 mm. The calibration of spatial inhomogeneities (flat-field response) for all detectors is performed using the fluorescence from a 10% HBr solution in a 3 mm capillary excited by an incident energy of 13.5 keV. The corresponding fluorescence emission is at 11.92 keV. To obtain good statistics, typically several hundred thousand counts per pixel are accumulated by multiple exposures. The efficiencies of detectors are estimated using SAXS intensity from deionized water (MilliQ) in the compressibility limit (1.6 × 10^−3^ mm^−1^ at 298 K) (Narayanan, 2014 ▸).

**Figure 6:**
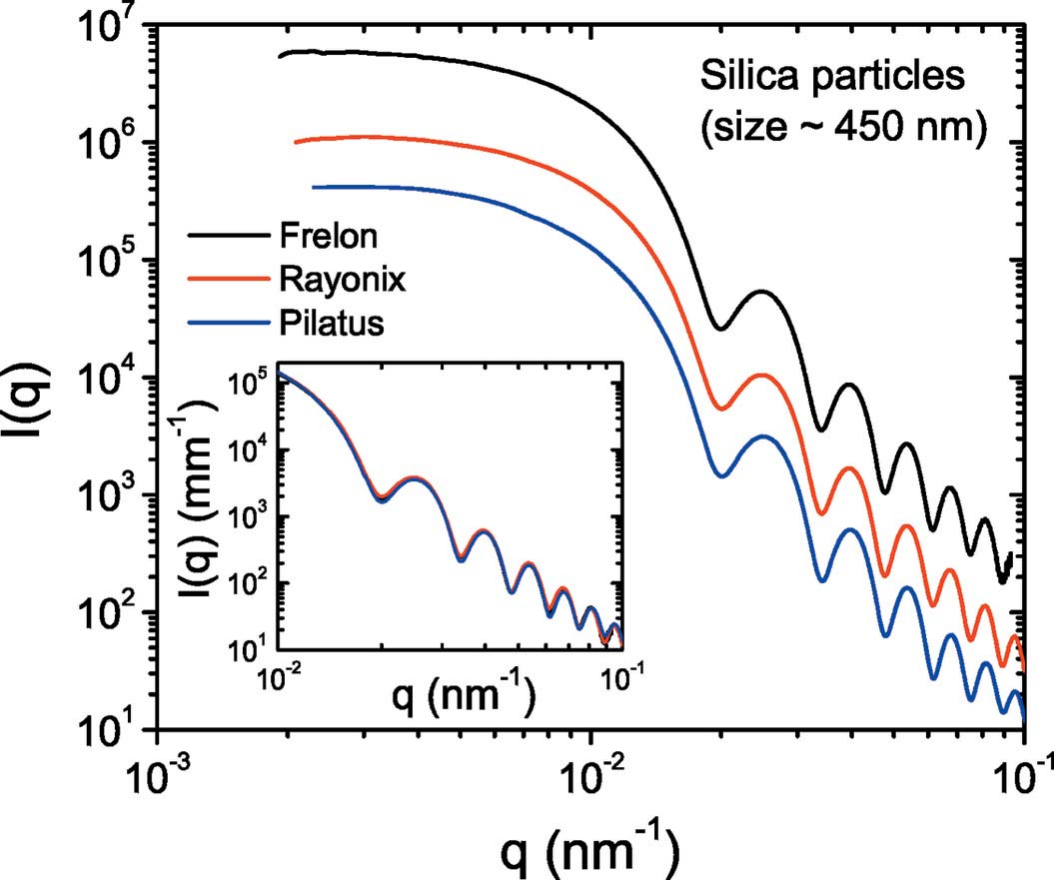
Azimuthally averaged scattering profiles of a dilute silica colloidal suspension recorded with different detectors at a sample–detector distance of 31 m. The first minimum corresponds to a mean radius of *R*_Mean_ ≃ 225 nm. The shifts in the intensities indicate the relative sensitivity factor of the detectors, which was not included in the normalization. The inset shows the data after normalization by this factor.

The spatial resolution of the detector is another important parameter which ultimately determines the resolution of the instrument. Fig. 6 ▸ displays the azimuthally averaged USAXS profiles of the silica particle suspension measured with different detectors. These profiles can be modeled by a polydisperse sphere scattering function with mean radius (*R*_Mean_) 226 ± 2 nm and polydispersity ∼5%. The oscillations in the intensities have the same amplitude, indicating that the detector spatial resolution is not affecting the measurement at this sample–detector distance (30.8 m) for this size and polydispersity of particles. Slight smearing of the first minimum in this case is due to multiple scattering (Semeraro, Möller & Narayanan, 2018 ▸) and not a resolution effect. The vertical shifts in intensities manifest the differences in the detection sensitivity, *i.e.* the number of detector counts in analog–digital units (ADU) produced per photon. The average sensitivity factors at 12.46 keV are 11.1, 2.7 and 0.56 for the FReLoN, Rayonix and Pilatus detectors, respectively. Once divided by these factors, all profiles superimpose as shown in the inset of Fig. 6 ▸. These factors indicate that for both CCD detectors the average single-photon level is above the mean pixel noise level, which is about 1.6–1.8 ADU. The Pilatus detector is a true photon-counting detector and the single-photon signal is discriminated from the noise.

### 3.1. High-brilliance SAXS and WAXS   

**Figure 7:**
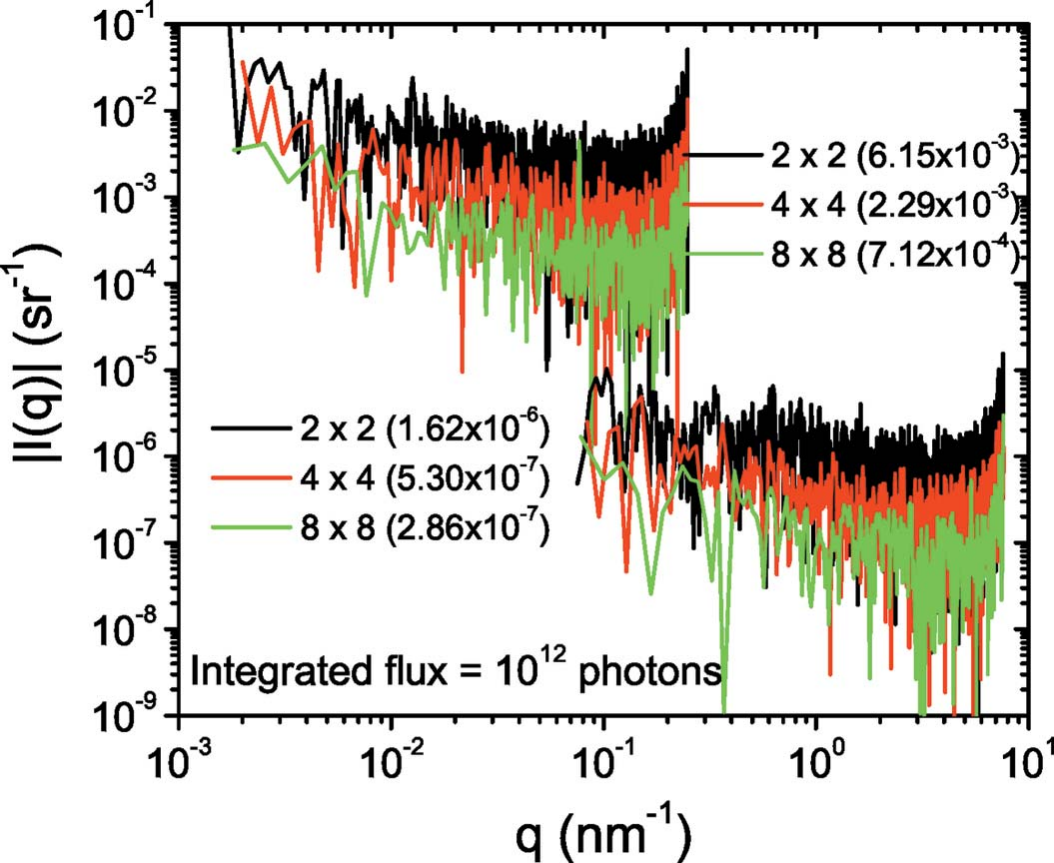
Detection limit of the Rayonix detector at 31 m (left) and 1 m (right) sample–detector distances for an incident flux of 10^13^ photons s^−1^ and an exposure time of 0.1 s (*i.e.* 10^12^ photons). For a comparison of the scale, the scattering by 1 mm of water is about 1.6 × 10^−3^ sr^−1^. The traces oscillate with respect to 0 and are therefore represented as their moduli.

The main applications of high-brilliance SAXS and WAXS are either probing kinetic processes or detecting weak structural signals. In radiation-sensitive samples, long exposure times cannot be afforded. The limit of detection is often set by the noise of the detector, and in addition the frame rate in kinetic studies and parasitic background at ultra-low angles. Fig. 7 ▸ illustrates the absolute detection limit, which is given by the residual dark counts in normalized intensity units, for the Rayonix detector at two extremes of the detector position. These difference dark patterns are measured by blocking all scattered photons on the detector while the intensity monitors are still receiving the indicated flux. The two-dimensional patterns were subsequently normalized and azimuthally averaged using the same procedure as in a SAXS measurement. The detection limit scales down with the solid angle subtended by the pixels since each pixel collects more photons for the same noise level. Similarly, it is inversely proportional to the total number of incident photons. The detection limit improves with hardware binning as each binned pixel collects more scattered photons for a noise level similar to that of an unbinned pixel, as evident in Fig. 7 ▸. Therefore, for the detection of weak signals and radiation-sensitive samples, maximum affordable hardware binning should be used. The corresponding curves of the WAXS detector are not shown since they lie roughly two orders of magnitude lower owing to the larger solid angle subtended. The FReLoN detector with a comparable pixel area (binned) has a lower detection limit as a result of the higher sensitivity factor. The traces in Fig. 7 ▸ are recorded without a sample and absorption in the sample scales up the detection limit proportionally. The scattering by water lies below the detection limit at 31 m sample–detector distance. Evidently, for weakly scattering samples where the count rate is not an issue, the Pilatus detector is better than the CCD-based detectors.

### 3.2. High-resolution SAXS and USAXS   

**Figure 8:**
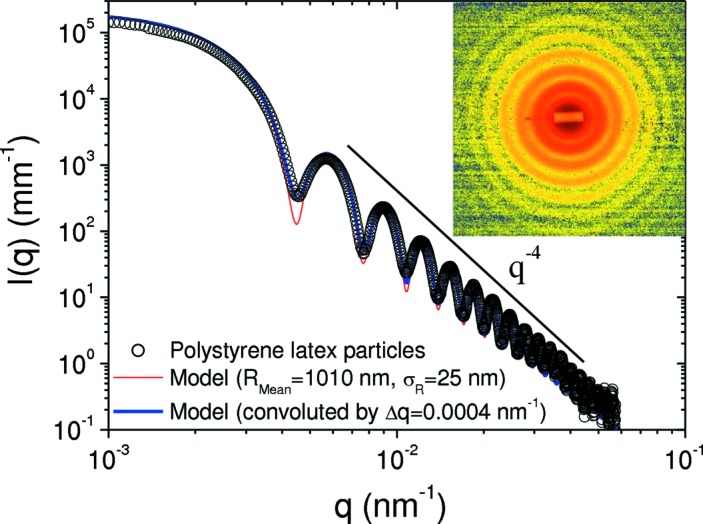
USAXS profile of polystyrene latex spheres (size ∼2 µm) in a water/ethanol mixture (1:3 by volume) recorded with the FReLoN detector at a sample–detector distance of 31 m. A Gaussian resolution function of FWHM Δ*q* ≃ 0.0004 nm^−1^ was used to convolute the model scattering function to fit the data (mean radius *R*_Mean_ = 1010 nm and standard deviation σ_*R*_ = 25 nm). The inset shows the corresponding background-subtracted two-dimensional scattering pattern.

Fig. 6 ▸ demonstrated that the effect of detector resolution is not significant for 0.5 µm particles (with 5% polydispersity) at 31 m sample–detector distance. The resolution effect becomes more pronounced at shorter distances with larger particles and lower polydispersities. This is illustrated in Fig. 8 ▸ using 2 µm size particles with 2.5% polydispersity. The normalized and background-subtracted *I*(*q*) cannot be fully described by a polydisperse sphere scattering function (mean radius = 1010 nm), and convolution by a Gaussian resolution function with FWHM 4 × 10^−4^ nm^−1^ is necessary to fit the data. In this way, the effective resolution of the setup can be determined in the absence of multiple scattering.

**Figure 9:**
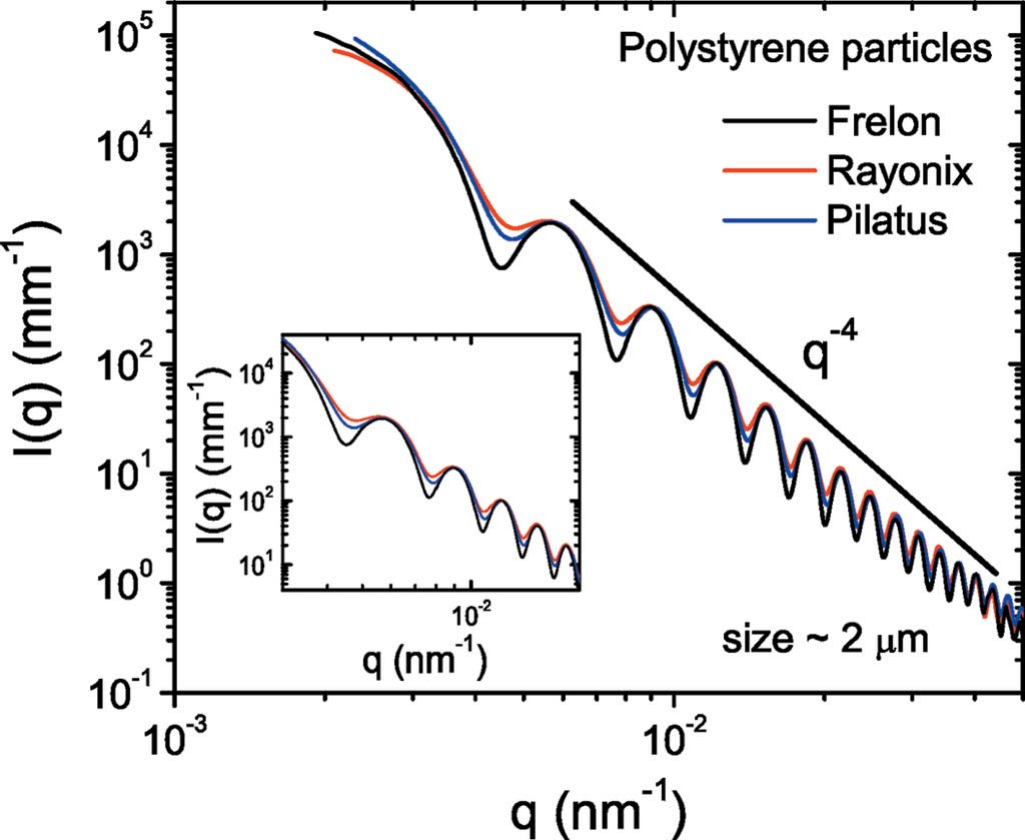
Comparison of the detector resolution visualized using the USAXS profile of polystyrene latex spheres (size ∼2 µm). All profiles can be described by the polydisperse scattering function shown in Fig. 8 ▸ convoluted by FWHM Δ*q* ≃ 4 × 10^−4^, 8.5 × 10^−4^ and 1.03 × 10^−3^ nm^−1^ for the FReLoN, Pilatus and Rayonix detectors, respectively.

A comparison of the data acquired with the other two detectors is shown in Fig. 9 ▸. Different degrees of smearing can be noticed for the three detectors, which can be described by the sphere scattering function convoluted by a Gaussian resolution function with the FWHM values indicated in the legend. The Pilatus detector has a better resolution than the Rayonix detector despite its much larger pixel sampling size (172 *versus* 44 µm). The smearing is primarily contributed by the detector PSF, which is nearly a box profile in the case of the Pilatus detector while the PSF of the Rayonix detector has a slowly decaying tail due to the phosphor response and imperfections in the fiber-optic taper (scattering and spatial distortion).

### 3.3. High resolution and coherence   

**Figure 10:**
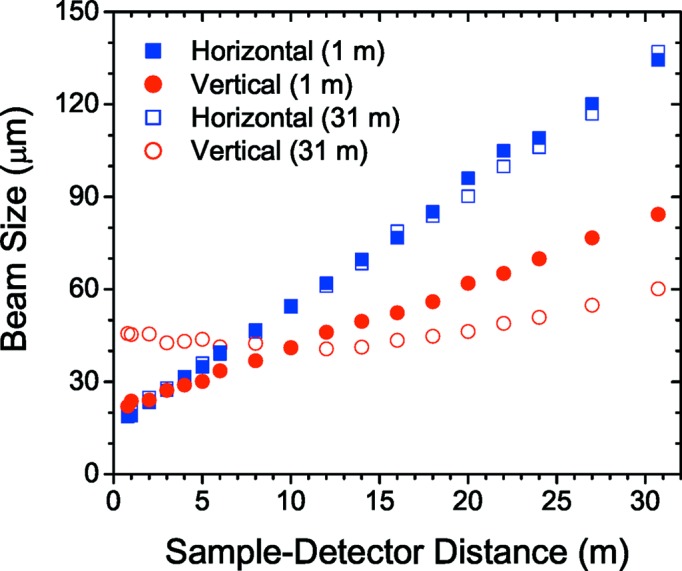
Minimum beam sizes obtained by slit collimation along the detector tube. The two sets of data correspond to mirror inclination optimized for the smallest size at the two extremities of the detector tube (1 and 31 m).

In order to further improve the resolution, a higher degree of collimation and smaller beam cross section are required. Fig. 10 ▸ presents the minimum (diffraction-limited) beam sizes measured using the beam viewer along the detector tube for the mirror inclination adjusted to focusing at the shortest and longest distances. In this configuration, the beam is nearly coherent (*i.e.* the beam cross section is comparable to the transverse coherence length) with a reduced flux of about 5 × 10^10^ photons s^−1^. Scattering patterns recorded with this beam display speckles as depicted in Fig. 5 ▸(*c*).

**Figure 11:**
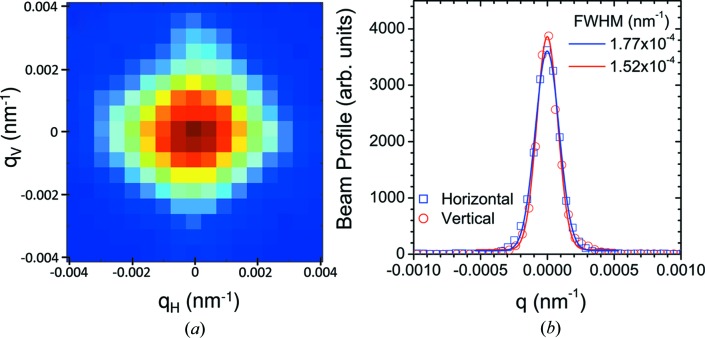
(*a*) High-resolution beam profile recorded with the FReLoN detector at a sample–detector distance of 30.8 m. (*b*) Corresponding horizontal and vertical profiles, which can be described by Gaussian functions with the FWHM values indicated.

Further reduction of the beam size can be obtained by offsetting the last secondary and first guard slits in opposite directions while maintaining the aperture above the diffraction limit. A nearly symmetric beam cross section at the farthest detector position can be obtained in this way. However, the flux is correspondingly diminished to about 10^10^ photons s^−1^. Fig. 11 ▸ displays the beam profile recorded at 30.8 m, which has near equal contributions from the detector PSF and the beam size. The FWHM limiting resolution is about 1.5 × 10^−4^ nm^−1^, that is a factor of 6 better than an Si 220 crystal-based Bonse–Hart instrument (Sztucki & Narayanan, 2007 ▸). In terms of angular resolution, this roughly corresponds to 2 µrad and the corresponding real-space resolution is about 50 µm (Petukhov *et al.*, 2015 ▸). Therefore, the actual spatial resolution probed in the scattering volume is now limited by the beam cross section rather than the angular resolution. The beam is highly coherent in this configuration, which is useful for XPCS measurements with strongly scattering samples. The optimum condition for high-resolution USAXS measurements is a beam cross section of 25 µm and angular resolution of 4 µrad, which is comparable to the resolution obtained with an Si 333 crystal.

### 3.4. Improvements with EBS upgrade   

**Figure 12:**
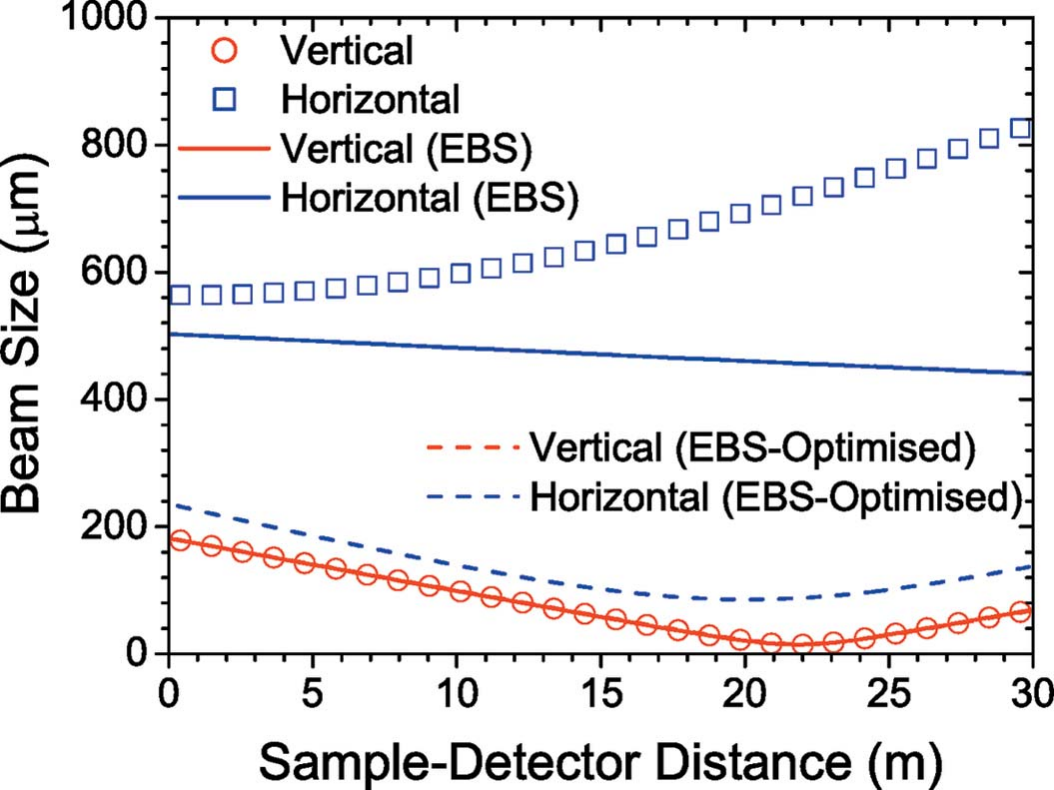
Comparison of the beam profiles in the vertical and horizontal directions with the present optics and source (symbols), after the ESRF-EBS upgrade (continuous lines), and with mirror specifications optimized for the EBS parameters (dashed lines). Note that the change along the vertical direction is not significant in the scale of the figure.

Here the expected performance with the ESRF EBS upgrade is briefly summarized. The main improvement with the upgraded storage ring will be in the horizontal direction where the beam size and divergence are expected to decrease by about a factor of 16 and 1.6, respectively. The change in the vertical beam parameters will not be significant. With the reduction of horizontal source size, the brilliance will be higher by a factor of 25−30 and correspondingly the coherent fraction of the photon flux will be roughly increased by a factor of 30. Nevertheless, the power density on the monochromator will remain the same. Fig. 12 ▸ depicts the calculated beam sizes for the present source and optics, for the EBS with the current optics, and with the mirror specifications optimized for the EBS properties. The horizontal beam improvement will be complemented by installing a high-performance pixel detector. Therefore, both USAXS and XPCS will greatly benefit from the EBS and the optimized optics. Further improvement of the beam in the future will be possible using a focusing and collimation scheme based on compound refractive lenses (Vaughan *et al.*, 2011 ▸).

## 4. Scientific applications   

This section describes some representative examples to demonstrate the potential of the instrument. More in-depth studies are reported in the corresponding references cited. One of the applications of a combined USAXS/SAXS instrument is to probe *in situ* the structural features from micrometre scale to nanometres (Beaucage, 2012 ▸). This aspect is illustrated in recent work on the ultrastructure of *Escherichia coli* bacteria under *in vivo* conditions (Semeraro *et al.*, 2017 ▸). USAXS and SAXS elucidated the structure from the whole cell to the ultrastructure of the cell membrane, providing complementary information to both optical and cryo-electron microscopies. Another notable example is an investigation of the hierarchical structure of microtubes formed by the self-assembly of an archetypical surfactant, sodium dodecyl sulfate (SDS), and a naturally abundant polysaccharide, β-cyclodextrin (Ouhajji *et al.*, 2017 ▸). In this case, the combination of SAXS/USAXS revealed the multiscale structure from their molecular capsids to folded microtubes. Here the molecular capsids organize to create rhombic two-dimensional crystalline bilayers which stack further to form multilamellar structures, and they subsequently fold to microtubes.

The scientific applications also critically depend on the availability of sample environments, and data analysis and modeling capabilities. Therefore, an array of sample environments is provided, which include rapid stopped-flow mixing (Narayanan *et al.*, 2014 ▸), a high-resolution rheometer (Panine *et al.*, 2003 ▸), and temperature and pressure jumps (Möller, Léonardon *et al.*, 2016 ▸). For data evaluation, relatively simple analysis and visualization features are implemented in the *SAXSutilities* package. More advanced modeling is performed using specialized program suits such as *SASfit* (Breßler *et al.*, 2015 ▸), *SasView* (Alina *et al.*, 2017 ▸) and *Irena* (Ilavsky *et al.*, 2018 ▸).

### 4.1. Broad-*q*-range time-resolved studies   

**Figure 13:**
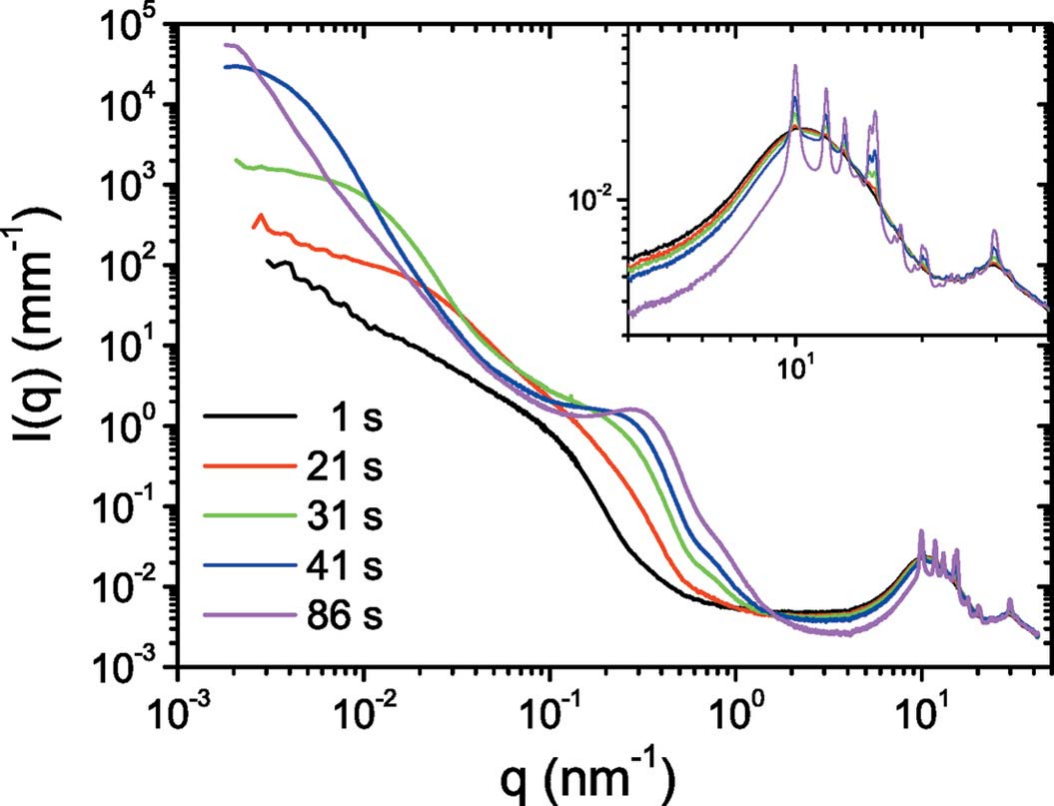
Time-resolved combined WAXS, SAXS and USAXS profiles during the isothermal crystallization of isotactic polypropylene from the melt at 418 K. The inset shows the evolution of WAXS intensities. The evolution of SAXS and WAXS profiles is analogous to that reported by Panine *et al.* (2008 ▸), but the USAXS region shows the development of large-scale structures.

One of the advantages of having access to a wide *q* range is the ability to follow the structural development at multiple length scales. A classic example is the crystallization of a polymer from the melt to form the semi-crystalline meso­structure, which further evolves to microscopic spherulite morphology. Fig. 13 ▸ displays the typical evolution of the SAXS and WAXS intensities during the isothermal crystallization of isotactic polypropylene. In this case, SAXS/WAXS and USAXS/WAXS data were acquired from two separate experiments under identical conditions. The crystallinity (deduced from WAXS) and the invariant, which is a measure of the mesostructure (derived from SAXS), follow the nucleation and growth behavior as found in previous work (Panine *et al.*, 2008 ▸). In addition, the USAXS region shows the formation of a large-scale structure which could be the precursor for the spherulite morphology. Development of this microscopic structure at relatively early stages of crystallization was not evident in the previous study using a Bonse–Hart instrument, presumably because of the higher background and time required to perform a USAXS scan, which was much longer than the times shown in Fig. 13 ▸. Qualitatively, these USAXS data may be interpreted in terms of the long-range density fluctuations at the initial stages, which subsequently develop into the well defined surface-fractal-like morphology of spherulites.

A more striking illustration is the recent study of the nucleation and growth of microtubes of SDS and β-cyclodextrin mentioned above, following a temperature quench from 348 to 298 K (Landman *et al.*, 2018 ▸). Time-resolved SAXS/USAXS experiments deciphered the structure development at different size scales and the underlying kinetics. The results allowed the identification of an inward growth mechanism of microtubes, which may be applicable to a variety of similar classes of systems.

### 4.2. Time-resolved USAXS   

Examples described in the previous section have already illustrated the utility of time-resolved USAXS for probing hierarchical structure development. A key strength of this method is in the investigation of optically opaque systems which cannot be easily observed under an optical microscope or by light scattering methods. In many synthetic systems, it may be possible to match the optical refractive index by choosing an appropriate solvent, thereby making the system less turbid. This approach is not feasible with protein solutions, most of which are opaque at high concentrations. Therefore, time-resolved USAXS has become a powerful tool for probing the early stages of liquid–liquid phase separation in concentrated protein solutions (Da Vela *et al.*, 2016 ▸). For example, the kinetics of spinodal decomposition in concentrated bovine serum albumin solutions undergoing liquid–liquid phase separation induced by trivalent ions was studied. The results revealed an arrested spinodal decomposition for deep temperature quenches into the liquid–liquid coexistence region (Da Vela *et al.*, 2016 ▸).

Another prototypical case is the coacervation of polyelectrolytes, which occurs instantaneously upon mixing oppositely charged polyelectrolytes, and the solution becomes very turbid. Time-resolved USAXS allowed probing of the millisecond scale assembly processes upon mixing anionic sodium polyacrylate and cationic polyallylamine hydrochloride at different salt (NaCl) concentrations (Takahashi *et al.*, 2017 ▸). Nearly charge neutral percolated clusters formed in the millisecond scale, which coalesced with time to form large agglomerates. At the same time, the morphology of the initially formed clusters became more compact by chain relaxation. In other words, the complexation is a multiscale process and time-resolved USAXS enabled deciphering of the kinetics at different structural levels.

### 4.3. High-resolution fiber diffraction   

**Figure 14:**
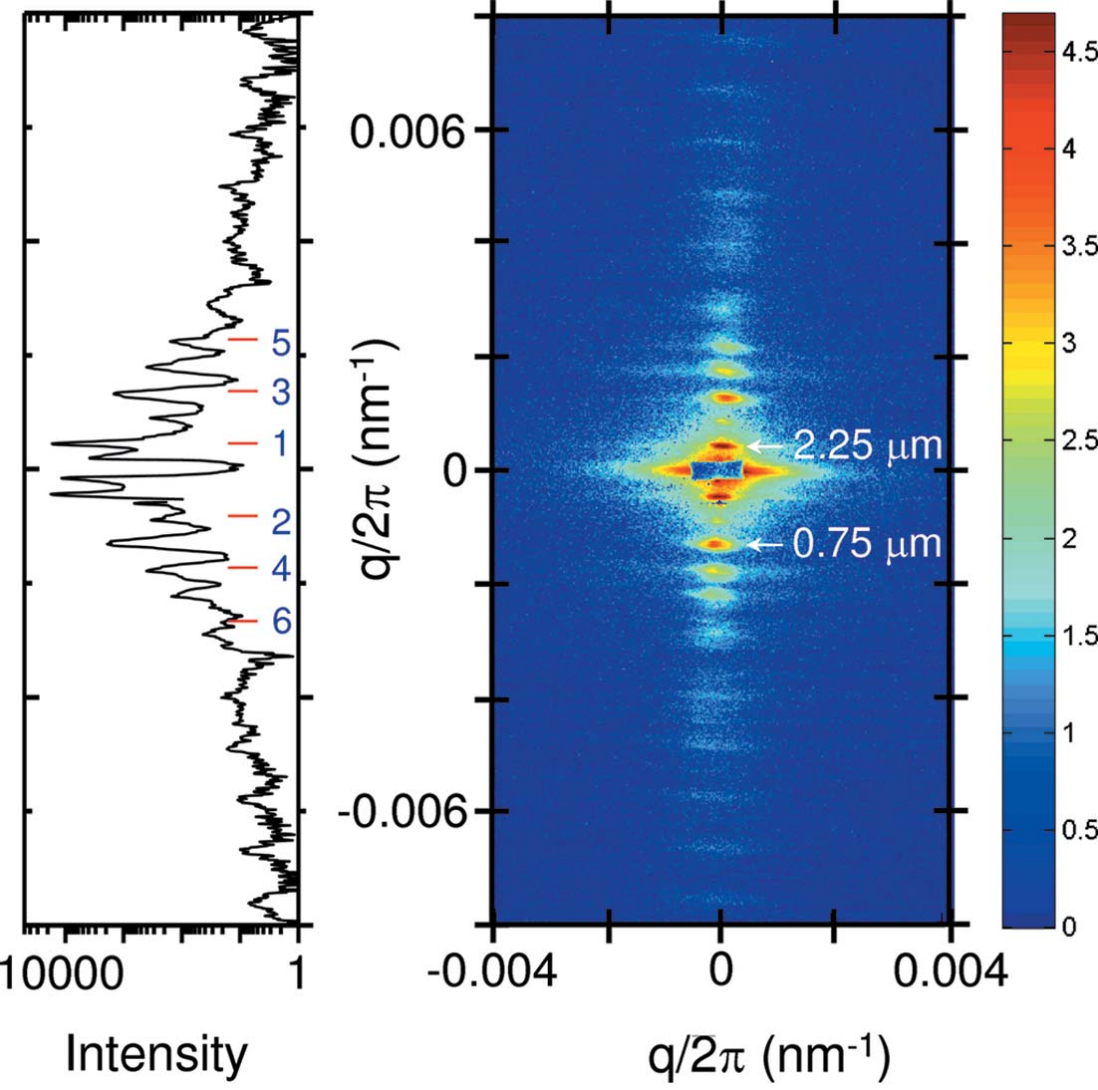
Ultra-low-angle diffraction pattern of a resting mammalian skeletal muscle manifesting the sarcomere reflections. The first-order peak corresponds to a sarcomere length of 2.25 µm. The intensity trace shown on the left is an integration over 10 horizontal pixels and indicates the successive diffraction orders. The specimen is courtesy of V. Lombardi *et al.* (University of Florence, Italy).

The small beam cross section, high degree of collimation and high-resolution detector greatly benefit fiber diffraction experiments. A well known case is structure–function studies in striated muscle (skeletal and cardiac), which until now have mainly focused on the structural dynamics at the level of filamental proteins myosin and actin. The long fiber–detector distance of 30.8 m permits accessing the structural changes at the sarcomere (muscle cell) level (Piazzesi *et al.*, 2018 ▸). Fig. 14 ▸ shows the background-subtracted ultra-low-angle diffraction pattern from rabbit skeletal muscle (extensor digitorum longus) in the resting state. The diffraction diagram clearly displays the lower-order peaks from the sarcomere which can be used to follow the changes at the cellular level during the contraction. This approach turned out to be a valuable tool for the investigation of the length-dependent activation in cardiac muscle (Reconditi *et al.*, 2017 ▸). The results provided compelling evidence for the mechanosensing role of the myosin filament as a second permissive step along the pathway of muscle regulation, supplementing the well established actin-based mechanism. The mechanosensing function facilitates high metabolic efficiency of striated muscle and explains the molecular basis for the Frank–Starling law of the heart (Piazzesi *et al.*, 2018 ▸).

One of the important assets for this type of experiments is the ability to investigate the structural changes at the level of filamental proteins as well as the sarcomere in the same preparation. The complementary information thereby derived significantly aids in interpreting the diffraction diagrams. Furthermore, these investigations undoubtedly will benefit from the upcoming EBS and having a high-resolution pixel array detector.

### 4.4. Ultra-small-angle XPCS   

Using the high degree of coherence obtained with tight collimation, XPCS experiments can be performed on samples with sufficient scattering power. USA-XPCS provides an easier access to the equilibrium dynamics in low-viscous media owing to the intrinsic slowing down at low *q*. Therefore, faster diffusive and advective dynamics in aqueous media can be probed by multispeckle XPCS without the need to manipulate the solvent viscosity (Möller, Chushkin *et al.*, 2016 ▸). This feature has already been exploited for the investigation of velocity fluctuations at the early stages of colloidal sedimentation (Möller & Narayanan, 2017 ▸), phoretic dynamics of colloids during the phase separation of the solvent (Dattani *et al.*, 2017 ▸) and active dynamics of Janus colloids (Semeraro, Dattani & Narayanan, 2018 ▸). The larger size of speckles at ultra-low angles allowed the use of a Pilatus detector for multispeckle XPCS measurements.

Recently, the performance of multispeckle USA-XPCS was significantly improved by using an Eiger 500k detector, which has a small pixel size (75 µm) and high frame rate (up to 22 kHz) (Zinn *et al.*, 2018 ▸). This allows a much broader range of experiments involving sub-millisecond dynamics. When this detector is used in combination with the EBS, it is expected that dynamics in many functional biological systems will become accessible.

## 5. Summary   

**Figure 15:**
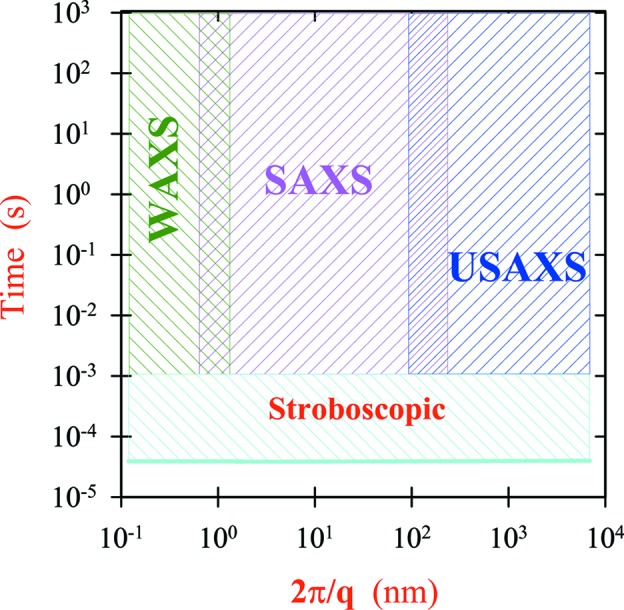
Nominal length and time scales accessible by USAXS, SAXS and WAXS techniques for a sample having sufficient structural features over the spanned range. Sub-millisecond experiments are performed stroboscopically using a tandem shutter scheme.

Following the upgrade, the ID02 beamline at the ESRF has evolved to a unique multipurpose time-resolved USAXS/SAXS/WAXS (TRUSAXS) instrument. The combined *q* range covered using 1 Å X-ray wavelength is about 0.001 ≤ *q* ≤ 50 nm^−1^, and *q* resolution down to 1.5 × 10^−4^ nm^−1^ can be obtained. Two-dimensional USAXS patterns from *q* < 0.001 nm^−1^ can be acquired, and the resulting one-dimensional profiles have superior resolution and statistics as compared to the same data measured with an optimized Bonse–Hart instrument. The overall size range covered by these techniques is from micrometre to ångström scales, and time resolution down to the sub-millisecond range can be achieved. Fig. 15 ▸ summarizes the length and time scales accessed by these techniques. In addition, the improved coherence properties of the beam can be exploited for performing USA-XPCS, which probes sub-millisecond dynamics at large length scales.

Despite the advances in different microscopy techniques, scattering methods remain essential for deriving quantitative structural and kinetic information in complex soft matter and biological systems. This impact is more significantly felt in the investigation of transient processes over millisecond time range and at large length scales in optically opaque systems. Further improvement of these methods is expected with the upcoming EBS upgrade, especially time-resolved SAXS and USAXS, and USA-XPCS will greatly benefit from the new source. The main applications of the instrument are in soft-matter and biophysical sciences, but it is also used for a broad range of industrial research and development.

## Figures and Tables

**Table 1:** Main specifications of the SAXS, WAXS and USAXS detectors for 12.4 keV X-rays The spatial resolution (FWHM) was measured using a 20 µm attenuated beam and fitting with a Gaussian profile. The maximum counts of CCD detectors are in analog–digital units (ADU) per pixel.

	Rayonix MX170	Rayonix LX170	FReLoN 4M	Pilatus 300K
	(SAXS/USAXS)	(WAXS)	(SAXS/USAXS)	(SAXS/XPCS)
Active area (mm)	170 × 170	85 × 170	50 × 50	84 × 107
Pixel size (µm)	44.2	44.2	23.8	172
FWHM resolution (µm)	85.4 ± 1.6	85.4 ± 1.6	44.3 ± 0.7	89 ± 2
Dynamic range (photons mm^−2^)	10^7^	10^7^	10^7^	3 × 10^7^
Maximum counts (ADU)	65 000	65 000	60 000	10^6^
Dark noise	< single photon	< single photon	<0.2 photon	Photon counting
Frame rate (s^−1^)	8 (2 × 2 bin)	8 (2 × 2 bin)	2 (1 × 1 bin)	400
	100 (10 × 10 bin)	100 (10 × 10 bin)	7 (8 × 8 bin)	
Integration time (ms)	≥1	≥1	≥1	≥0.2
